# Crowd flow forecasting via agent-based simulations with sequential latent parameter estimation from aggregate observation

**DOI:** 10.1038/s41598-022-14646-4

**Published:** 2022-07-01

**Authors:** Fumiyasu Makinoshima, Yusuke Oishi

**Affiliations:** Fujitsu Limited, Kawasaki, 211-8588 Japan

**Keywords:** Natural hazards, Civil engineering

## Abstract

Unlike conventional crowd simulations for what-if analysis, agent-based crowd simulations for real-time applications are an emerging research topic and an important tool for better crowd managements in smart cities. Recent studies have attempted to incorporate the real-time crowd observations into crowd simulations for real-time crowd forecasting and management; however, crowd flow forecasting considering individual-level microscopic interactions, especially for large crowds, is still challenging. Here, we present a method that incorporates crowd observation data to forecast a large crowd flow, including thousands of individuals, using a microscopic agent-based model. By sequentially estimating both the crowd state and the latent parameter behind the crowd flows from the aggregate crowd density observation with the particle filter algorithm, the present method estimates and forecasts the large crowd flow using agent-based simulations that incorporate observation data. Numerical experiments, including a realistic evacuation scenario with 5000 individuals, demonstrated that the present method could successfully provide reasonable crowd flow forecasting for different crowd scenarios, even with limited information on crowd movements. These results support the feasibility of real-time crowd flow forecasting and subsequent crowd management, even for large but microscopic crowd problems.

## Introduction

Over the past few decades, various crowd simulation models have been developed^1^, and the utilisation of the crowd simulation has rapidly increased in our society. For example, crowd simulation techniques have been actively employed to assess evacuation safety for various hazardous events, such as fires^2^, floods^3^ and tsunamis^4^. In addition, crowd simulation has also been utilised in designing environments^5^ and has other diverse applications^6^.

Along with the development of simulation techniques, recent developments in tracking technologies, such as video analysis, provide richer observation data regarding crowds^7^. Recently, such analyses have been further enhanced by novel techniques, such as machine learning^8^. Additional data from various observation channels, such as GPS, Wi-Fi and Bluetooth, are also useful to understand the crowd movements^9,10^. The number of such observation channels is increasing in the recent smart cities, where information communication technologies are being pervasively installed and utilised to improve various activities^11,12^, and the available data for a more realistic crowd simulation is growing and becoming exceedingly rich. By utilising richer observation data, crowd simulation models or parameters can be calibrated based on empirical data^13–16^; however, the validity of such identified parameters in different or future events should be examined. As an example, an empirical study^17^ reported a clear difference in evacuation response tendencies for different sociocultural groups. Although crowd simulation is a powerful tool for various crowd-involved applications, it is mostly limited to what-if analysis under the given scenarios, which is mainly caused by the uncertainty in real crowd movements. This challenge limits the use of crowd simulations for real-time applications, such as real-time crowd forecasting and management. Data from recently enhanced observations with AI and IoT are increasing and can be available in real-time; thus, the utilisation of such rich observation data for agent-based models in real-time may exceed the conventional what-if analysis and enable real-time crowd forecasting based on real-time observation data. Such real-time crowd flow forecasting is imperative in smart cities as it can provide vital information to mass event organizers or authorities for better crowd managements for improved mobility and crowd disaster prevention^18^.

Utilising real-time data to make simulations “live” is an important but challenging topic in the field of agent-based simulations^19^; recent studies have attempted to develop techniques to incorporate observation data into agent-based models for more accurate simulations. For macroscopic crowd flows, Sudo et al.^20^ proposed the city-scale real-time human mobility estimation method using an extended particle filter and tested it against the data collected on the day of the 2011 Great East Japan Earthquake. Their network-based model estimated large-scale human mobility in road networks in an area of approximately 68 km$^2$ every 30 s, which was better than baseline models. Lueck et al.^21^ developed the particle-filter estimator using sparse population counting sensors for tracking crowds in ongoing events, and verified the proposed method against simplified evacuation scenarios on a simple network model including 100 agents. Their numerical experiments confirmed the performance of the estimator under different sensor configurations. Rife et al.^22^ further confirmed whether the estimator^21^ could track the individual-level behaviour from the aggregate population counting data and discussed its potential to extract individual identity from aggregate sensor data. Cai et al.^23^ proposed using an agent-based model in a network and particle filter with low-cost population data to reconstruct human mobilities in cities. The developed method was applied to a local city, covering approximately 669 km$^2$, and the prediction performance was validated using mesh population distribution and traffic census. Although these studies have proposed a method applicable to large crowd flows, their network-based macroscopic models have a limitation in providing sufficient crowd details (e.g., individual-level interactions), which is vital for managing and preventing crowd disasters.

The number of existing studies is limited; however, some have attempted to incorporate observation data into microscopic crowd simulations, which can consider detailed individual-level interactions. Wang and Hu^24^ proposed the use of a particle filter with observation data from sparse binary proximity sensors to estimate the two-dimensional positions of individuals as a likely initial condition for more accurate simulations. Their method was tested by tracking up to 6 individuals, and the result showed that the estimation became difficult as the number of individuals increased due to an increase in the complexity of the system to be estimated. Malleson et al.^25^ investigated the performance of the particle filter algorithm to incorporate position observations into a simple two-dimensional microscopic agent-based crowd model (*StationSim*) to locate the positions of individual agents. They tested the method against scenarios including up to 40 agents. The results showed that the performance of the estimation became worse as the number of agents increased; this was because the behaviour of the agents diverged due to their increased interactions. Clay et al.^26^ similarly tested the applicability of the unscented Kalman filter to a two-dimensional simple crowd problem with *StationSim* , and the proposed method was verified against scenarios involving up to 30 agents with limited observation conditions. Although the result indicated the potential applicability of the proposed method to larger crowds with limited information, the increased difficulty of estimating larger crowds was also confirmed in the experiments.

As reviewed, despite the increasing attempts to develop techniques for incorporating crowd observations into agent-based simulations, there are still challenges for their practical applications. In particular, the number of people considered in the simulation is still limited to a small population (e.g., tens of people with a microscopic model), and the interactions among agents are rather simplified. As a result, crowd forecasting for large crowds with detailed agent-based models remains challenging, mainly because of its complexity induced by microscopic interactions. Additional efforts in developing methodologies for real-time detailed crowd flow forecasting are required to realise better crowd management in smart environments.

Here, we propose a data assimilation method to estimate and forecast relatively large but microscopic crowd flows (e.g., thousands of people) by introducing the concept of latent parameters. Given that aggregate crowd observations such as density maps are available, the present method assumes the latent parameters behind the crowd, which macroscopically govern the observed crowd behavioural tendency. Subsequently, both the state of the crowd and the latent parameters can be sequentially estimated from the given observation using the particle filter algorithm. In numerical experiments, we demonstrate that microscopic agent-based crowd simulations with sequentially estimated latent parameters successfully provide reasonable estimates of the crowd flow, even for microscopic large crowd movements involving thousands of individuals.

## Results

### Crowd flow forecasting framework

**Figure 1:**
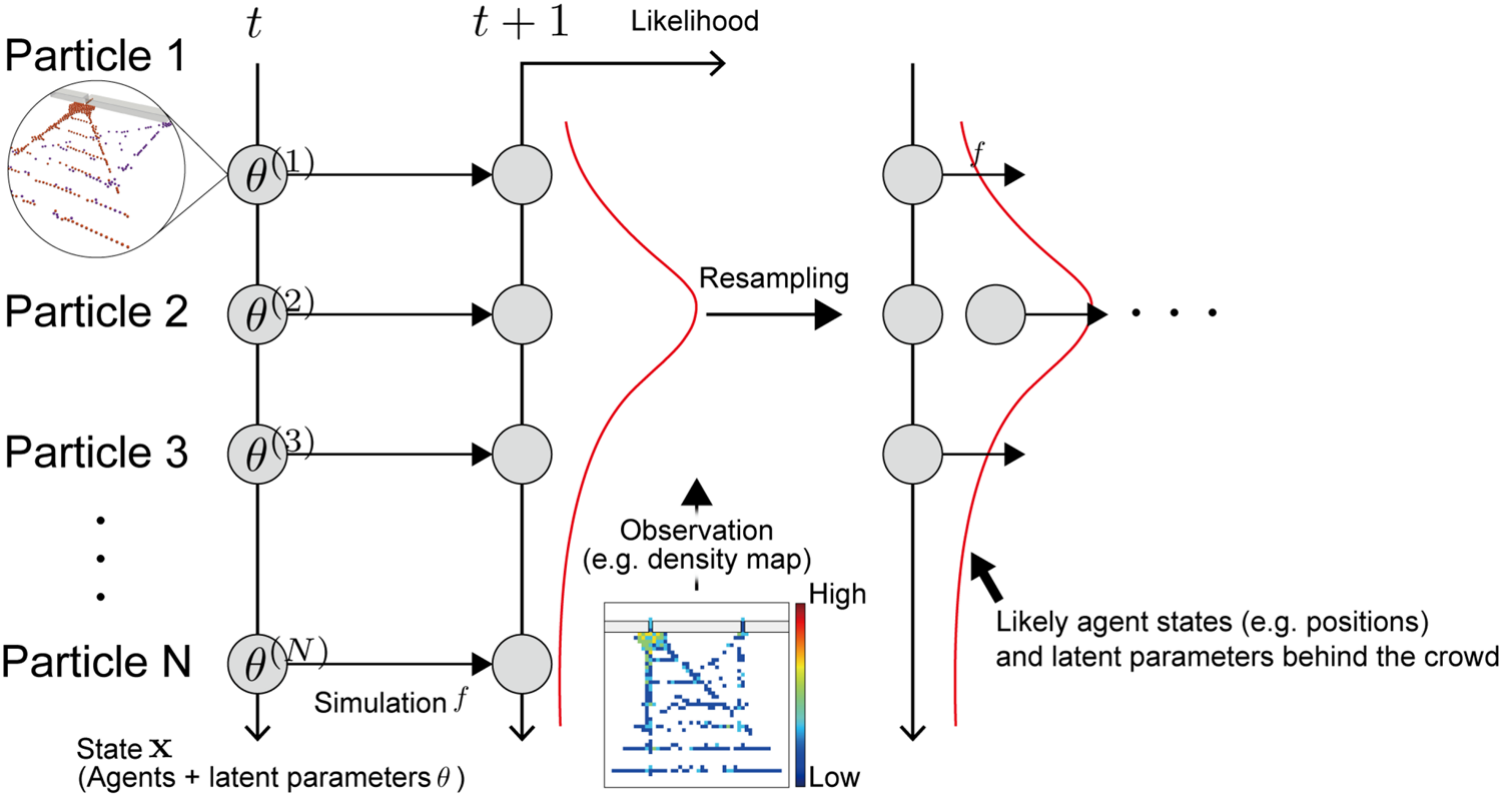
Schematic illustration of the proposed crowd forecasting approach. By sequentially estimating the likely crowd states and latent parameters behind the crowd flow based on observation data, the present method provides better crowd flow forecasting.

Figure 1 visualises the schematic illustration of the proposed crowd flow forecasting approach. In the present method, we assume that some primary simple behavioural modes exist behind real complex crowd flows, and complex crowd flows emerge from the superposition of the behavioural modes. For example, although individual circumstances or intentions vary, complex crowd flow at a train station might be explained by the superposition of behaviours based on choices from different platforms. If we can successfully estimate the composition of the behavioural modes in real-time, the crowd simulation with the estimation should give better crowd flow forecasting while reducing the complexity of the simulation model.

According to the above concept, we prepare a crowd simulation model equipped with the parameters $\varvec{\theta }$ that are assumed to exist behind the target crowd flow and control the composition of the primary behavioural mode. In this study, we refer to the parameters $\varvec{\theta }$ as latent parameters. The present method uses the simulation models with different latent parameters as particles within the particle filter algorithm, as shown in Fig. 1. Because crowd simulation with different latent parameters can result in various crowd flows, we obtain various possible crowd states by running the forward simulations. Then, at the point when real-time crowd observation (e.g., aggregate density observation from cameras) can be obtained, we can evaluate the particles and estimate the likely crowd flows and latent parameters. The actual composition of behavioural mode is not observable from aggregate observations alone; however, assuming that a better fit between the model and observations indicates a higher consistency of the considered latent parameters, we can estimate the likely composition of behavioural modes as latent parameters through agent-based simulations and particle filter algorithm. By sequentially estimating the likely crowd states and latent parameters behind the crowd flow, the present method provides better crowd flow forecasting using real-time observation data. For details of the simulation model and the procedures of the latent parameter estimation, see the “Methods” section.

### Synthesising crowd flow through exits

To verify the proposed forecasting approach, we synthesised the crowd flows using a force-based crowd simulation^4^, which can simulate individual-level microscopic interactions among agents and obstacles. For details of the force-based agent-based simulation, see the “Methods” section. The synthesised crowd flows were prepared to be used as observations for crowd forecasting, and the forecasting performance was verified against them, that is, the identical twin approach adopted in the previous studies^24,25^.

**Figure 2:**
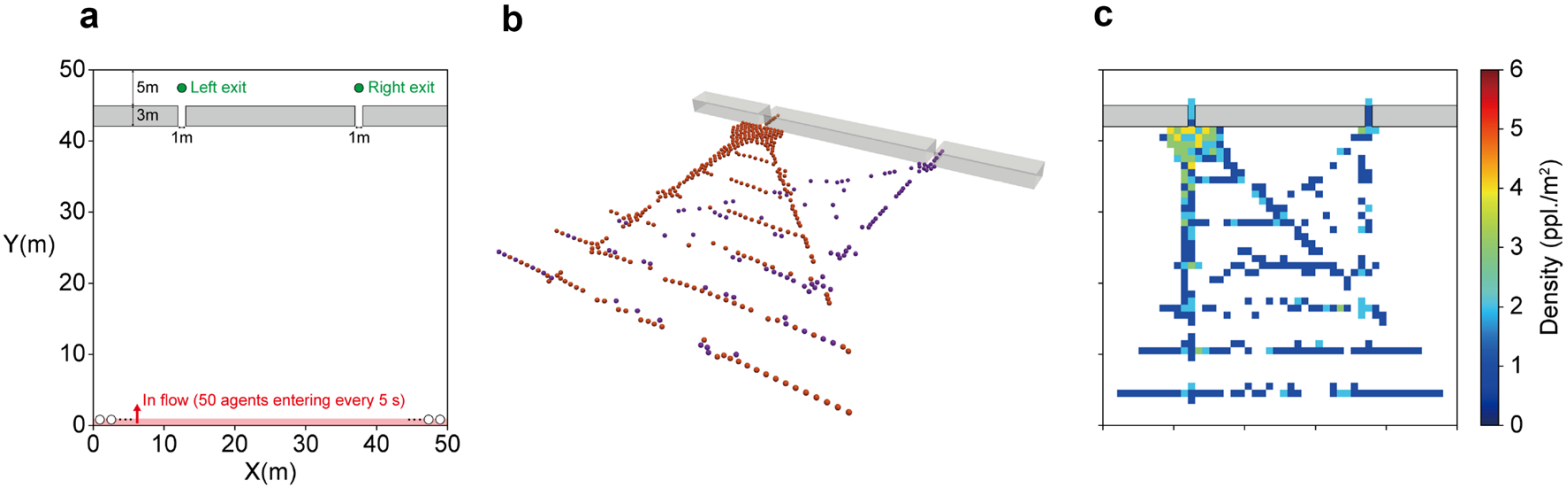
Model setup and synthesised crowd for Experiment 1. **a** Configuration of the environment. **b** Synthesized crowd flow. The purple and orange particles represent agents who chose the right and left exits, respectively. **c** Assumed corresponding density observation to be obtained.

The first synthetic observation data were obtained by simulating the crowd flow passing through simple exits (hereafter called Experiment 1). Figure 2 summarises the simulation setup and the synthesised crowd flow. The simulation environment for Experiment 1 had two exits, and a total of 1000 agents entered the environment at a given rate (50 agents every 5 s) from the bottom (Fig. 2a). In contrast to the commonly employed nearest exit choice assumption, in which the number of agents choosing the right and left exits becomes approximately equal, we assumed that the agents had a certain preference for the exits, which better mimics a realistic crowd flow. In this experiment, the ratio of agents choosing the right and left exits was set to 3:7. This preference rate was constant during the simulation, i.e., the behavioural tendency was time-invariant.

Corresponding to the assumed exit preference, a crowd flow concentration at the left exit and the resulting congestion can be observed (Fig. 2b). In this study, we considered that the density map was available as an observation of crowd flow (Fig. 2c). As shown in the figure, the density map used in this study represents the number of pedestrians in grids ($1 \times 1$ m$^2$), which cannot distinguish individuals. Recent video analysis can easily detect the number of people in a certain area, and the aggregate observation result is privacy-aware; thus, the use of crowd density as an available observation is a reasonable assumption for practical use in terms of technical and privacy aspects. Consequently, only the entry rate of the agents and the density observation were assumed to be available for crowd forecasting in Experiment 1. Therefore, the present forecasting method must estimate the assumed exit preference from the aggregate observations for an accurate forecasting.

### Synthesising evacuation crowd from a beach

**Figure 3:**
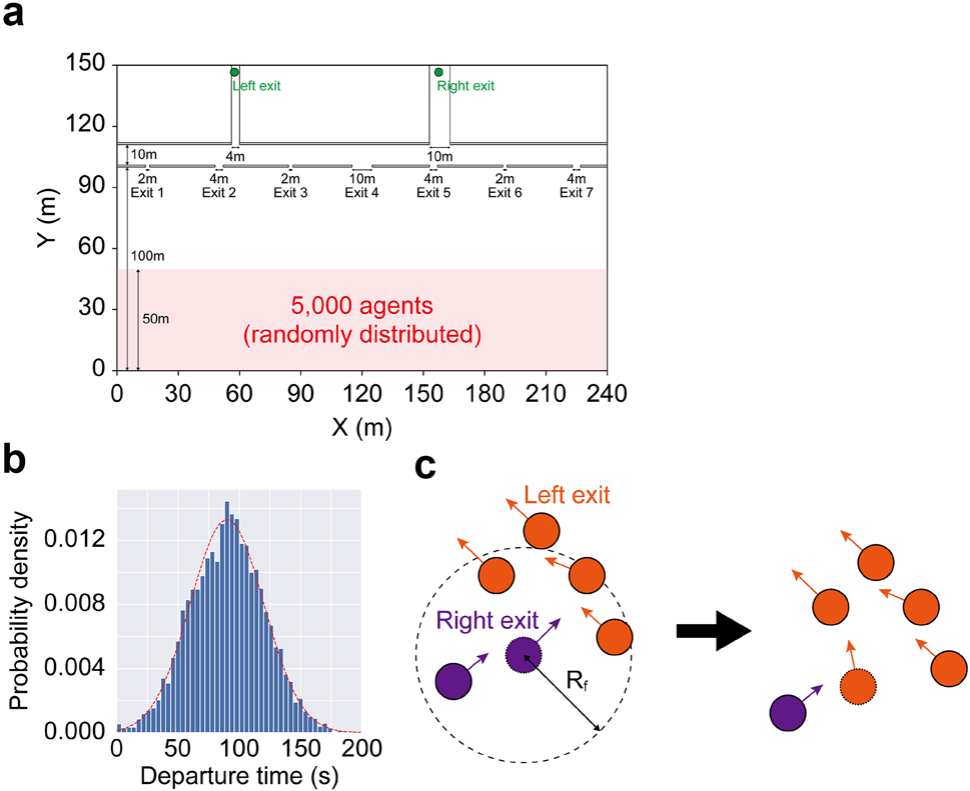
Model setup of Experiment 2 (evacuation). **a** Configuration of the environment. **b** Assumed departure timing. The synthesised departure timing for 5000 agents is visualised as a histogram with the assumed theoretical distribution (red dashed line). **c** Schematic view of the follower agent model. At each time step, the follower agents search for neighbouring agents moving within $R_f$ and change their exit preference by following the local majority.

The second synthetic observation assumed a more complex crowd flow: an evacuation from a beach (hereafter called Experiment 2). The simulation environment for Experiment 2 was based on an evacuation simulation model for the Miura beach in Japan^27^ (Fig. 3a). Although the model was based on a specific beach, similar structures can be seen on other beaches in Japan^27^. The environment had seven exits (Exits 1 – 7) with bottleneck widths ranging from 2 to 10 m, and the following two exits reached the right and left exits. As for the initial location of agents, we randomly distributed 5000 agents in a $50 \times 240$ m$^2$ area. In contrast to Experiment 1, we considered two factors for simulating the more complex behaviour of the agents.

The first was the evacuation departure distribution, as shown in Fig. 3b. The evacuation departure timing was based on a normal distribution $\mathcal {N}(90,30^2)$. The agents in the simulation checked the simulation time every 1 s and started to move if the simulation time exceeded their departure timing.

The second was the introduction of follower agents in the simulation. A notable characteristic of evacuation from beaches is the involvement of visitors. Previous studies that surveyed actual evacuation preferences for a potential tsunami at beaches revealed that most people at a beach are visitors, and thus are not familiar with the surrounding area^28,29^. In addition, the survey also reported that the behaviours of such visitors tended to depend on the surrounding people^28^. This empirical evidence suggests that the tendency of the evacuating crowd at beaches can change drastically according to the number of following evacuees, resulting in a significant uncertainty that makes forecasting difficult. To model this nature in synthesising realistic behaviours, we considered a simple follower model and included 4500 follower evacuees in the simulation. Figure 3c shows a schematic view of the simple follower model. At each time step, follower agents searched for surrounding agents within $R_f$ (10 m) and change their destination by following the local majority within $R_f$. When the follower agents could not find three or more individuals around them, they chose the nearest exit. The non-follower agents were considered to randomly select the right or left exit with an equal probability.

**Figure 4:**
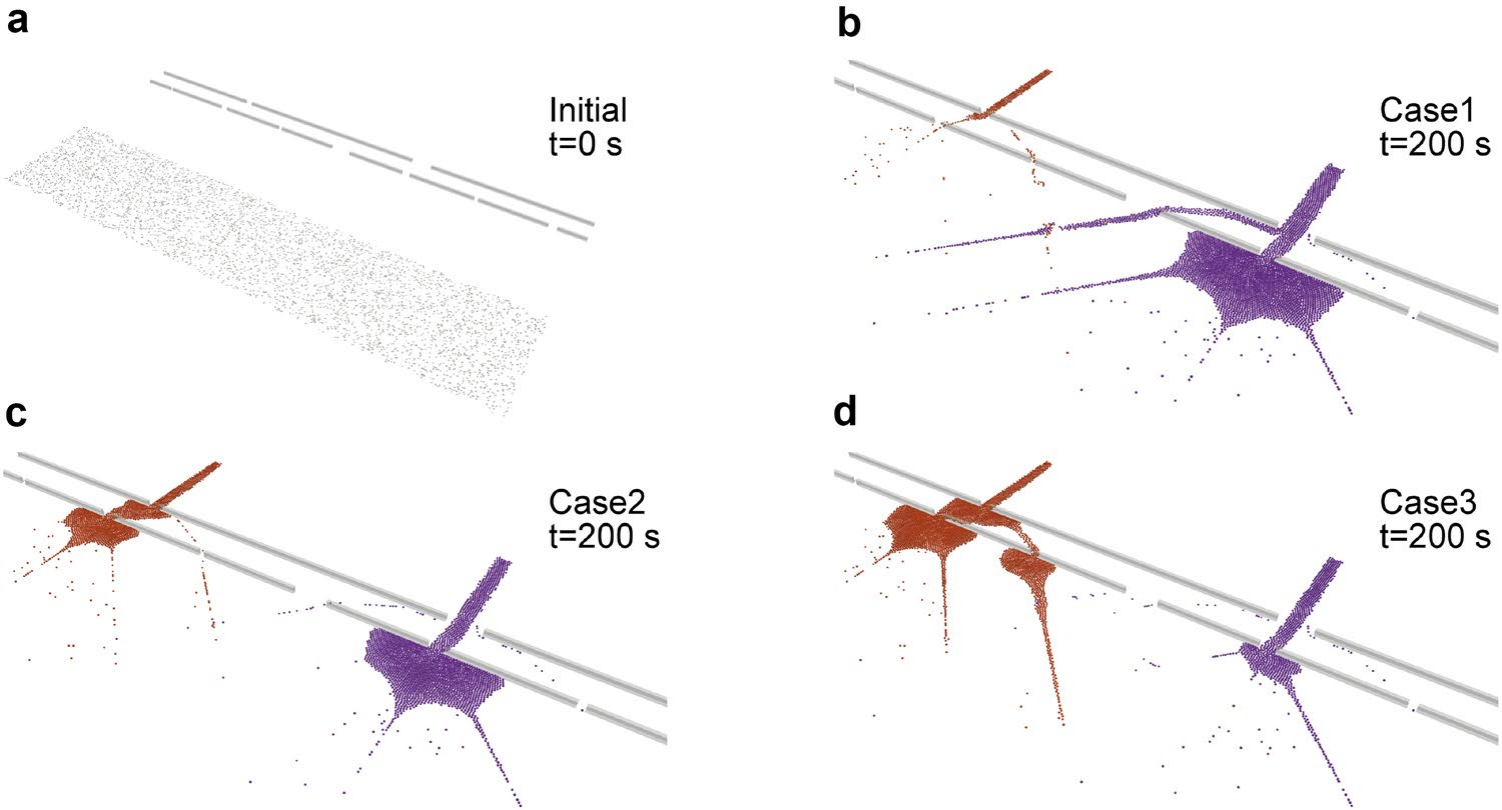
Synthesised crowd flow in Experiment 2. Three cases of the synthesised crowd flow at $t=200$ s are visualised with the initial agent distribution at $t=0$ s. The orange and purple particles represent agents who chose the left and right exits, respectively. **a** Initial state of the evacuees. **b** Evacuating crowd at $t=200$ s in Case 1. **c** Evacuating crowd at $t=200$ s in Case 2. **d** Evacuating crowd at $t=200$ s in Case 3. The randomness of the exit choice in a small fraction of non-follower agents with a large number of following agents can cause a significantly different evacuation flow tendency, which is difficult to predict via what-if simulations in advance.

The simulated crowd largely diverged because of the randomness in the exit choice of the non-follower agents and the effect of follower agents. Thus, we ran 500 simulations and selected three unique cases to verify the forecasting method: Case 1 (marked the largest number of evacuees for the right exit among 500 runs); Case 2 (experienced distributed evacuees at the right and left exits and marked fastest evacuation completion among 500 runs); and Case 3 (gathered the largest number of evacuees at the left exit and recorded the longest evacuation completion among the 500 runs). Note that the choice of the exit for non-follower agents was the only random factor in the simulation, and other factors such as departure timing and initial position were fixed in this synthesis. Figure 4 summarises the initial setup of the simulation at $t=0$ s and the simulated crowd for the three distinctive cases at $t=200$ s. The simulations demonstrated that different levels of congestion at the exits could be caused and even enhanced by the small uncertainty in the behaviours of agents. This indicates the difficulty in forecasting evacuating crowds because of the uncertainty in both the evacuation departures and the behaviours of visitors. If we observe the $T_{90}$, the time required to achieve an evacuation completion rate of 90% that is often used as an important indicator for evacuation time estimates in various evacuation problems, such as in nuclear accidents^30^, a difference in evacuation delays corresponding to the congestion tendency can be observed (374 s in Case1; 338 s in Case2; and 448 s in Case3). Such potential congestion and the corresponding delay in evacuation from beaches have been also suggested in an actual evacuation drill at a beach^31^. Therefore, evacuations from a beach are valuable case studies to test the capability of the present forecasting method because of the significant uncertainty in crowd movements and the potential usefulness of crowd flow forecasting, which can lead to real-time evacuation guidance for smoother evacuations. As a result, in Experiment 2, the performance of the present forecasting method was verified by investigating whether it could estimate the evacuating crowd with observations in which only the initial position of agents at $t=0$ s and the snapshots of the aggregate density map were available. A clear difference compared to Experiment 1 is that the behavioural tendency in Experiment 2 is time-variant; thus, the performance of the present forecasting method for dynamically changing behaviours can be confirmed in Experiment 2.

### Forecasting simple crowd flows through exits

We first verified the present method against the simple crowd flow through exits (Experiment 1). For Experiment 1, observations within 50 s were considered available, and subsequent crowd movements were forecasted using agent-based simulations with the estimated state and a single latent parameter $\theta _{1}$, which was assumed to control the exit preference. For a detailed setup of the latent parameters for Experiment 1, see the “Methods” section.

**Figure 5:**
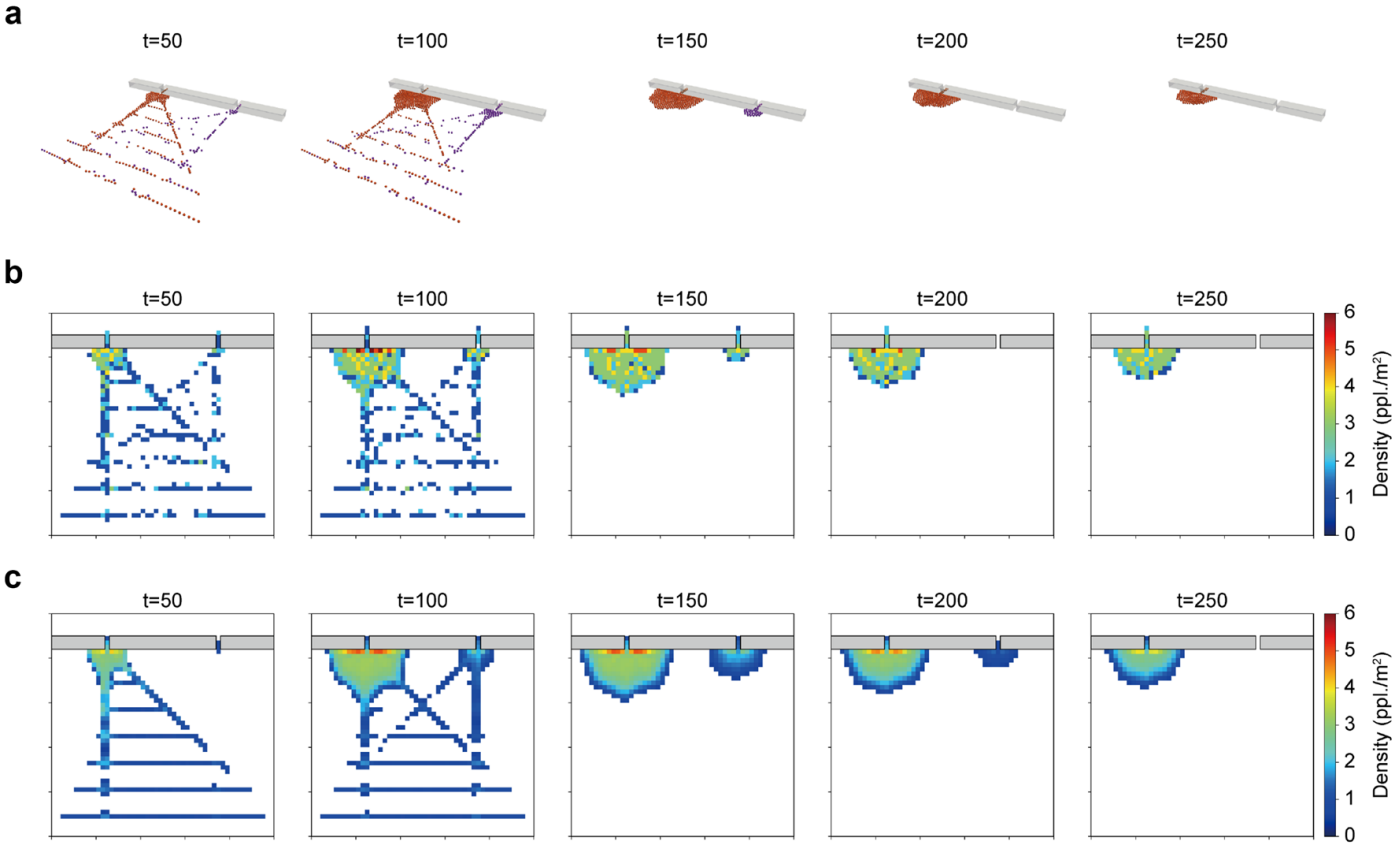
Comparisons between the ground truth simulation results and the forecasting results. **a** Ground truth simulation results. **b** Ground truth density maps (corresponding observations). **c** Estimated density maps (forecasting). Note that the estimated density value was visualised as an NaN colour (white) if the rounded value was lower than one.

**Figure 6:**
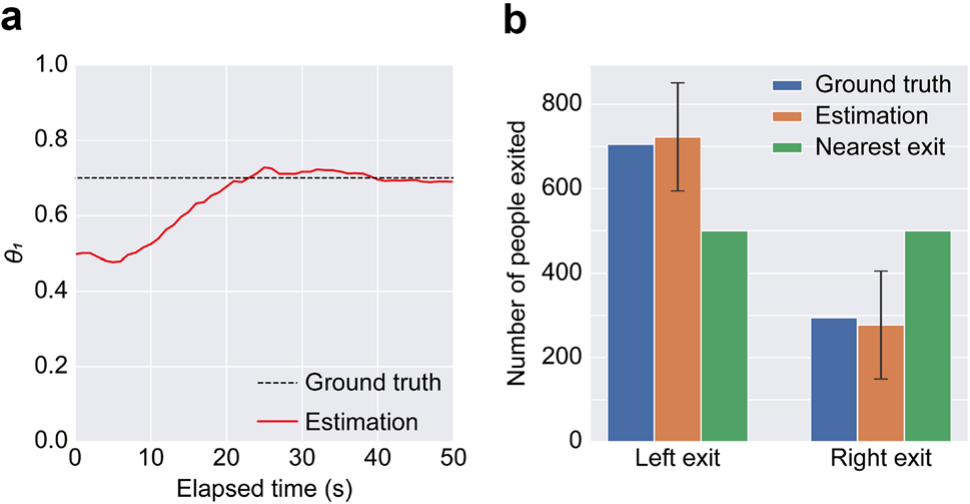
Estimated parameter and the resulting statistics. **a** Time series of the estimated parameter $\theta _{1}$. The black dashed line represents the assumed parameter (0.7) when synthesising the observation. The estimated parameter $\theta _1$ is visualised as a solid red line. **b** Comparisons of the cumulative number of people for the right and left exit. The error bars for the estimation represent the standard deviations.

Figure 5 compares the ground truth simulation results (Fig. 5a, b) and the estimated crowds (Fig. 5c). As shown in Fig. 5c, the method successfully estimated the crowd concentration at the left exit. Although crowding at the right exit was slightly overestimated at the later phases ($t=150$ s, 200 s), the level of congestion (e.g., extent of the crowd) at the left exit agreed with the ground truth. Within the observation period (50 s), only half of the total agents could be observed; however, the method gave a reasonable estimation of crowd flow. Figure 6a shows the time series of the estimated $\theta _{1}$ value with the assumed exit preference rate in crowd synthesis. At the earliest phase, the probability of choosing the right and left exit was roughly equal ($\theta _{1}\sim 0.5$) because there was not enough available observation to infer exit preference. As the observations increased, a clearer behavioural tendency could be observed, and the method estimated that the left exit was likely to be preferred by the crowd. The assumed preference rate of 0.7 in the synthesis was successfully estimated after approximately 20 s, and a consistent estimation of $\theta _{1}\sim 0.7$ could be observed in subsequent periods. Since the crowd flow in Experiment 1 had a time-invariant behavioural tendency, the method successfully estimated the subsequent crowd flow, as confirmed in Fig. 5c, even observing the part of the considered agents. As a result, both the behavioural process and the resulting number of people exiting both exits were accurately estimated, while the crowd simulation with the conventional nearest exit choice assumption failed to predict the imbalanced exit preference (Fig. 6b).

### Forecasting evacuating crowd flows

The present method was further verified against complex evacuating crowd flows at a beach (Experiment 2). For Experiment 2, only the initial position of agents and the snapshots of the density maps were considered available, and crowd movements with time-dependent departures and exit preferences including the realistic following behaviours were forecasted using agent-based simulations with the estimated state and two latent parameters, $\theta _{1}$ and $\theta _{2}$, which were assumed to control the exit preference and evacuation departure tendency, respectively. For a detailed setup of the latent parameters for Experiment 2, see the “Methods” section.

**Figure 7:**
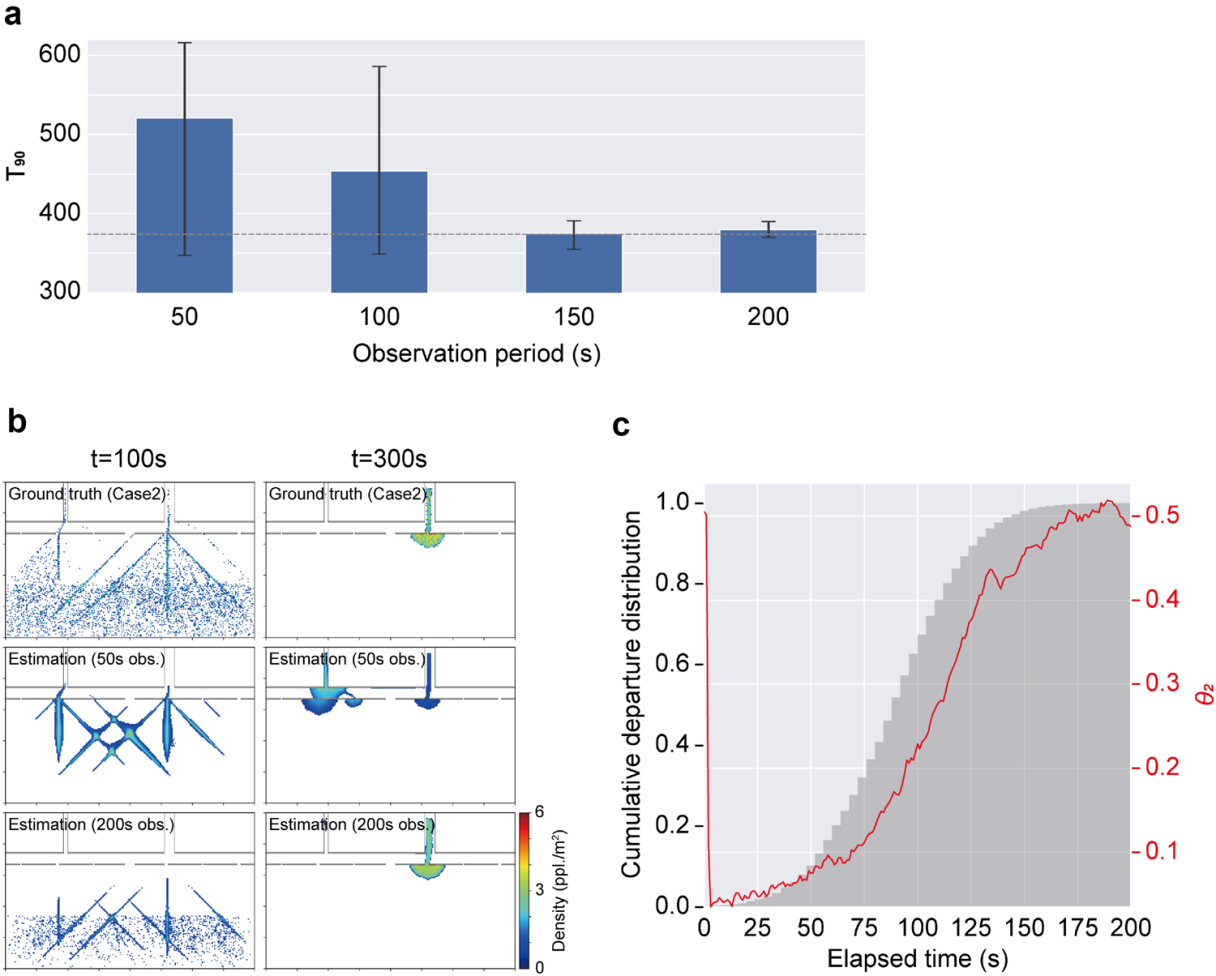
Crowd flow forecasting results for Case 1. **a** Estimation of $T_{90}$ with different observation periods. Error bars represent the standard deviation of the estimation. The grey dashed line represents the ground truth value. **b** Comparison of the estimated crowd flow at $t=100$ s and 300 s. Ground truth data are visualised in the top panel. **c** The time series of the estimated $\theta _{2}$ with the 200 s observation. The red line represents the estimated $\theta _{2}$. The cumulative departure distribution is shown as a grey histogram for reference.

The crowd flow in Experiment 2 is rather complex compared to Experiment 1, i.e., the synthesised crowd has a following behaviour that the estimator does not have, and the crowd tendency is time-variant because of the considered departure distribution. Hence, the performance of the forecast with different observation periods was first confirmed using the observation data of Case 1. To verify the proposed method in the Experiment 2, we evaluated the estimated time required to achieve an evacuation completion rate of 90% ($T_{90}$) as well as the estimated crowd flow. Figure 7a compares the estimation of $T_{90}$ for different observation periods. With the shorter observation period (e.g., 50 s and 100 s), the $T_{90}$ values were overestimated with relative errors of 39.3% and 21.4%, respectively, and the variation of the forecast was large, as shown by the error bars. The subsequent longer observation periods improved the estimation results, i.e., relative errors of 0.3% and 1.6% for 150 s and 200 s observations, respectively, and the estimation results converged, as can be observed by their smaller variation.

To understand where the estimation error for the shorter observation period arose, Fig. 7b visually compares the estimated crowd flow with 50 s and 200 s observation periods to the ground truth at $t=100$ s and 300 s. In the ground truth at $t=100$ s, an obvious crowd flow moving towards the exits can be observed while many individuals remain. However, the estimation with 50 s observation failed to estimate the timing of evacuation, and the estimation showed large moving crowds. In contrast, the estimator with 200 s observation successfully regulated the departure tendency and reproduced the slower departure tendency. At $t=300$ s, in the ground truth, crowds experienced congestion near the right exit, and almost no evacuees were found on the left side. The estimator with 50 s observation period failed to estimate not only the timing of the evacuation but also the exit preference of crowds. This result confirmed that both the misestimation of the timing of evacuation and the exit preference led to the large errors found in Fig. 7a. With sufficient observation, the estimator successfully estimated the crowd flow at $t=300$ s, which was almost equivalent to the ground truth.

Figure 7c shows the time series of the estimated value of the latent parameter $\theta _{2}$ with 200 s observation to confirm how the estimator with longer observation produced the distributed evacuation departures. In this figure, the cumulative departure distribution of the ground truth data is visualised as a reference. At the very beginning, no information is available to determine $\theta _{2}$ since the detailed crowd movement cannot be detected in the density map with coarse resolution. As soon as the estimator realises from the observed density map that estimations with large $\theta _{2}$ are unlikely, the estimated $\theta _{2}$ drops to nearly zero. Subsequently, the estimated $\theta _{2}$ gradually increases corresponding to the evacuation departure in the ground truth. As a result, in contrast to the conventional simulations in which evacuation departure tendency was given in advance based on previous observations or assumptions, the developed method could estimate the actual ongoing evacuation departure tendency from the observations. The estimator only knows snapshots of the density maps as observations, and no information regarding the assumed departure curve is given; however, the present method successfully provided reasonable crowd flow forecasting by sequentially estimating the state of the crowd and the latent parameters. These results demonstrated that the present method can be applied to crowd flows with time-variant behavioural tendencies.

**Figure 8:**
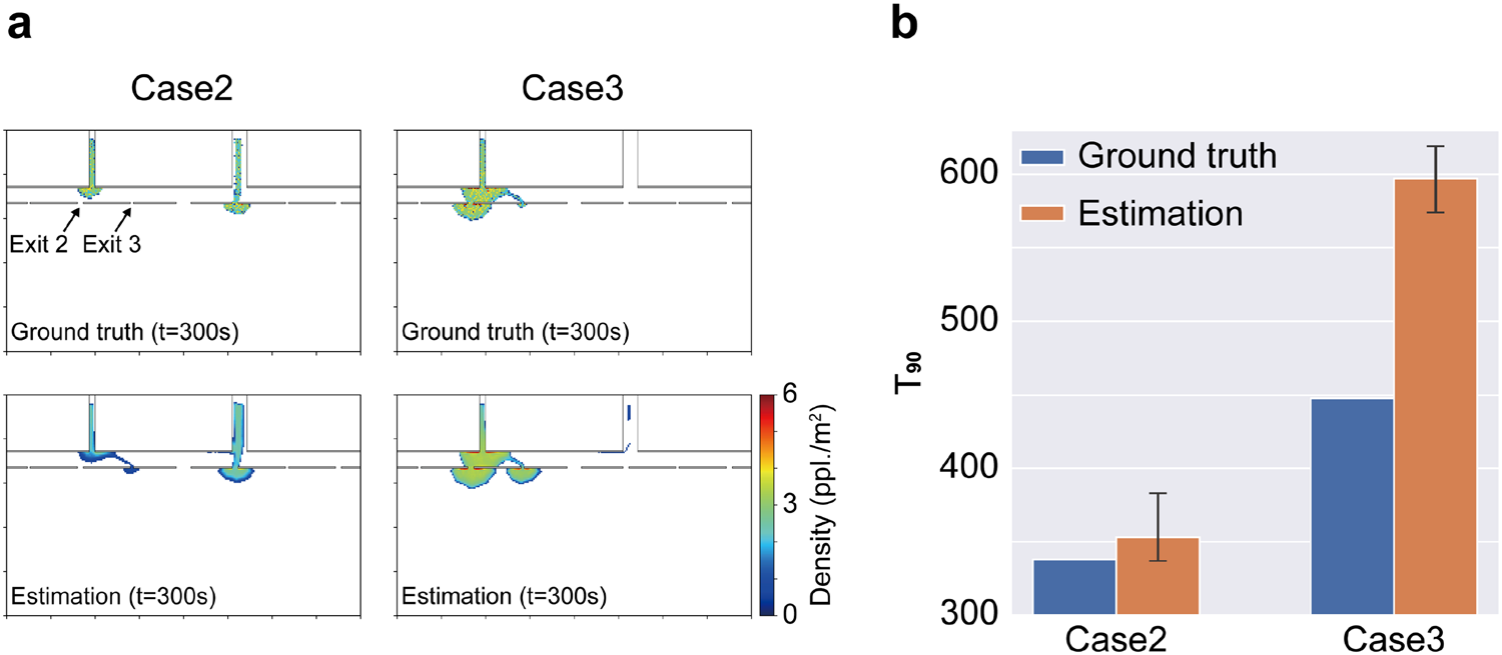
Forecasting results for Cases 2 and 3. **a** Comparison of the crowd flow between the estimation and the ground truth data at $t=300$ s. **b** Comparison of the $T_{90}$ value between the estimation and the ground truth.

**Figure 9:**
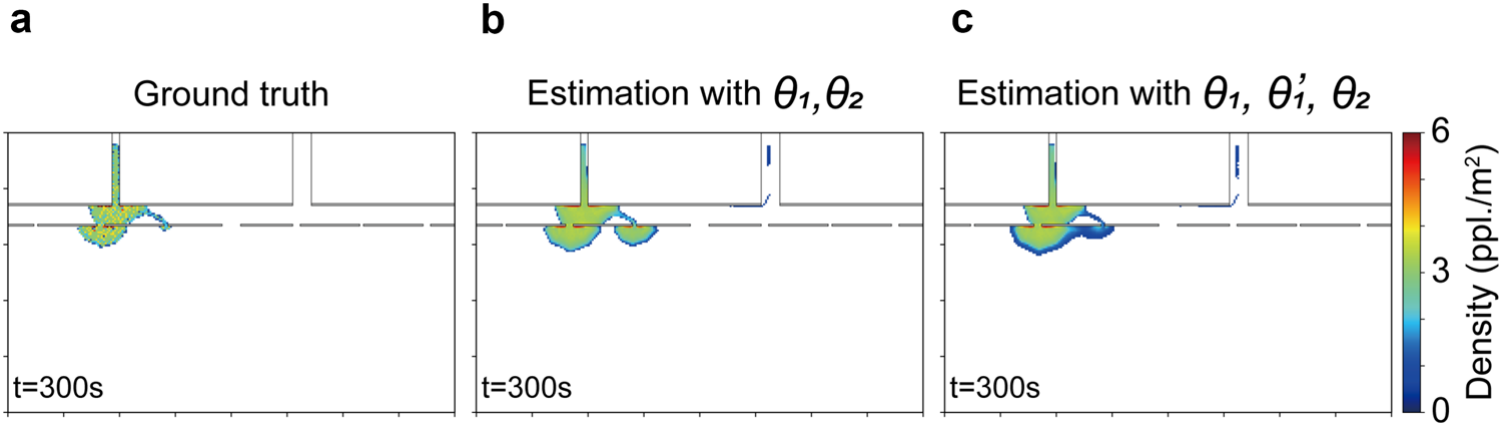
Forecasting results with a different number of latent parameters and the ground truth data at $t=300$ s. **a** Ground truth observation. **b** Forecasting result with two latent parameters. **c** Forecasting result with three latent parameters.

To further verify the applicability of the present method, we applied the same algorithm to different crowd flows (Cases 2 and 3). Owing to the fact that the above results for Case 1 indicated that a 200 s observation period was sufficient in Experiment 2, further performance investigations for the rest of this study were conducted with a 200 s observation period for comparison. Figure 8 summarises the estimation results for Cases 2 and 3. Some disagreements between the estimation and the actual flow, mainly caused by the simplification in the estimator (i.e., the realistic following behaviour was not included in the estimation, and only the macroscopic behavioural tendency was controlled by the latent parameters), could be found; however, the general behavioural tendencies were successfully estimated. For Case 2, a characteristic distributed evacuating flow could be estimated (Fig. 8a), and the resulting $T_{90}$ was also estimated with reasonable accuracy (a relative error of 4.4%). The same algorithm successfully forecasted the distinctive feature of the crowd flow in Case 3 (e.g., the crowd concentrating at the left exit); however, the congestion at Exit 3 was overestimated. Even with a sufficient observation period, this overestimation caused a large error in the estimated $T_{90}$ value, which reached a relative error of 33.3%, as shown in Fig. 8b. The main cause of this error arose from the gap between the complex real (synthesised) crowd movement and the simple crowd model considered in the estimator. In the synthesised crowd flow, the following behaviours were introduced to synthesise realistic behaviours, and this effect caused an imbalance of concentration among exits. In contrast, the movements of the agents towards the exits in the estimator were only modelled by the exit selection (right or left) with the single parameter $\theta _{1}$, and the agents tried to follow the shortest path towards the selected exit. As a result, the single parameter was not sufficient to accurately produce the imbalance between Exits 2 and 3 while reproducing the overall congestion level. To verify this explanation, we included an additional latent parameter $\theta _{1}^{\prime }$ to be estimated, which controlled the finer exit preference between Exits 2 and 3 after selecting the left exit. Specifically, two different navigation maps were created for the left exit by changing the cost for passing through Exits 2 and 3, and these maps were included in the exit selection. The estimation result with the additional parameter for Case 3 is shown in Fig. 9, with the ground truth and the original estimation. The result demonstrated that the additional latent parameter produced an imbalance between Exits 2 and 3 and improved the estimation result. These results confirmed that only a few parameters were required to forecast relatively complex microscopic crowd flows if the latent parameters were appropriately designed. With more latent parameters, more complex behaviours could be considered in the estimator; however, at the same time, the required number of particles in the particle filter to produce a reliable estimate would also increase. Thus, in practical applications, the considered latent parameter can be adjusted depending on the forecasting accuracy required for target applications.

### Forecasting with limited observations

The results in the previous sections show that the present method can provide reasonable crowd flow forecasting by utilising snapshots of the density map. The performance was verified with the full observation of the density map; however, in reality, such observations are limited by the observation equipment. For example, such a density map can be obtained by analysing observations from a surveillance camera that has a limited field of view (FOV). To verify the performance of the forecasting method assuming a more realistic situation, we ran the estimation again for Case 1 but with density maps within a limited FOV. In this experiment, we assumed two different camera installations (Setups 1 and 2) with various FOVs, and the error for weighting the particles was evaluated only within the FOV, i.e., the errors outside the FOV did not affect the estimation. In contrast to the original full observation, direct information regarding the congestion at the exits was not available in the limited observation with the FOV.

**Figure 10:**
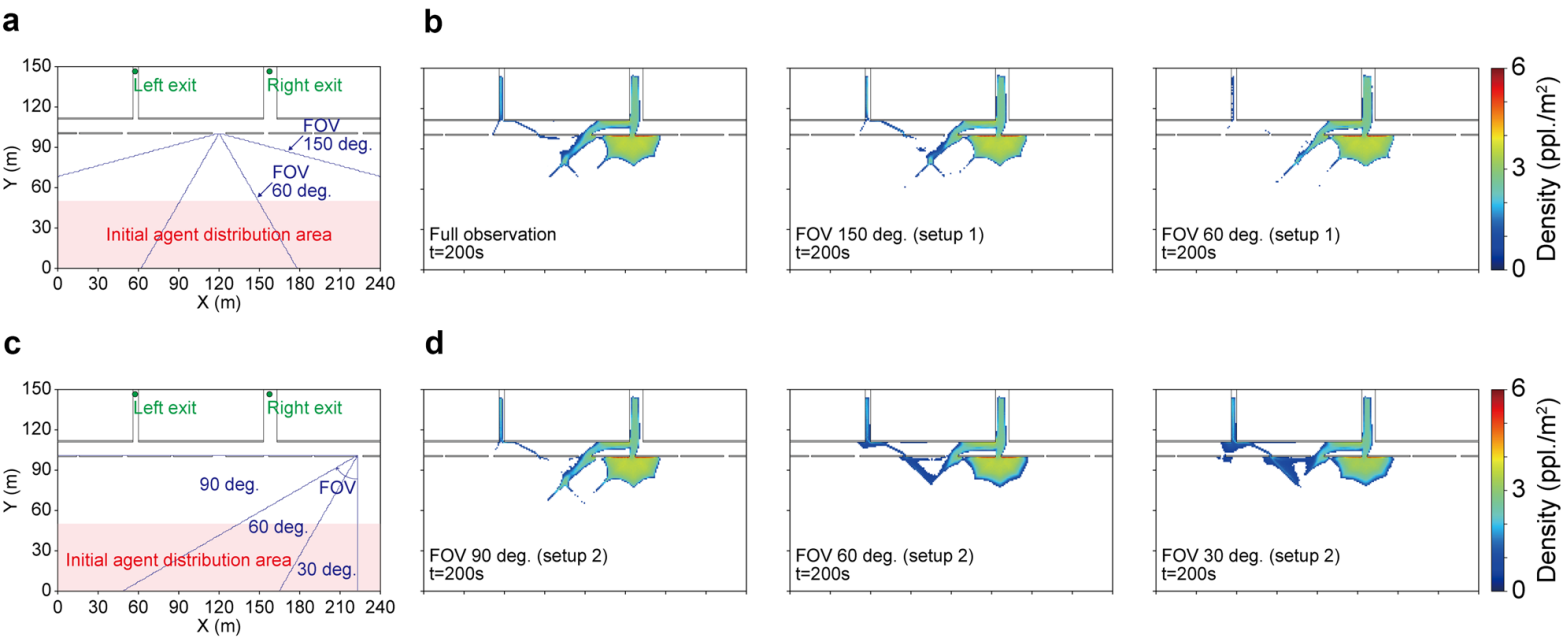
Crowd flow forecasting with limited observation settings. **a** Schematic view of Setup 1. The blue lines represent the assumed field of views (FOVs) for the limited observations. **b** Estimated crowd flows with the full and limited observations in Setup 1. **c** Schematic view of Setup 2. The blue lines represent the assumed FOVs for the limited observations. **d** Estimated crowd flows with limited observations in Setup 2.

In Setup 1, the camera was placed at the centre of Exit 4, and two different FOVs ($60^{\circ }$ and $150^{\circ }$) were assumed (Fig. 10a). Figure 10b presents the estimation results with full observation and limited observations in Setup 1. As shown in the figure, even with the limited density map, the estimation results are almost equivalent to the results with full observation and agree with the ground truth. A possible reason for this successful estimation is that a large portion of crowd flows towards the right exit (e.g., as seen in the ground truth crowd flow in Fig. 7b) can be observed even with the limited FOVs in Setup 1. Thus, different camera setups that cannot capture the primal crowd flows will lead to poorer estimations.

To further examine the performance of the method with limited observations, we additionally prepared Setup 2 (Fig. 10c), in which a camera having different FOVs ($30^{\circ }$, $60^{\circ }$, and $90^{\circ }$) was installed at Exit 7. In contrast to Setup 1, the information regarding crowd flows on the left side is not available from the camera with narrower FOVs in Setup 2. Figure 10d shows the estimation results obtained in Setup 2. While the observation with $90^{\circ }$ FOV resulted in a good estimation equivalent to the result with full observation, the estimation performance became worser with narrower FOVs, as expected. Although the congested crowd at Exit 5 could be estimated even with narrow FOVs, the large crowd flow towards the right exit through Exit 4 could not be estimated with observations from narrower FOVs, resulting in the overestimation of the crowd towards the left exit. These results can be explained by the feature of Setup 2, i.e., almost no information regarding crowd flows from the left side was available, and only the partial crowd flows towards Exit 5 were observed with the narrow FOVs. These results with limited observations indicate that direct or full observation is not always necessary for reasonable crowd forecasting, and even a part of the behavioural observation is sufficient if the observed behaviours have enough information to infer the behavioural tendency of the target crowd flow.

When we focus on the cover ratio (the ratio of the number of observable grids to the total number of grids) of the setups, we find that a higher cover ratio does not necessarily lead to a better estimation performance. For example, although the estimation with FOV $60^{\circ }$ in Setup 1 was more consistent with actual crowd flow than that with FOV $60^{\circ }$ in Setup 2, the cover ratio of the former setup was smaller, i.e., the cover ratio of $60^{\circ }$ in Setups 1 and 2 were 16.2% and 24.5%, respectively. This result implies the importance of the optimal observation equipment installation for target crowd flows and the potential of accurate crowd flow forecasting with limited but optimised observations.

## Discussion

In this paper, we presented a crowd flow forecasting method that sequentially estimated both the crowd state and latent parameters from the aggregate density observation data. While previous studies using data assimilation techniques with microscopic agent-based models^24–26^ attempted to locate individual behaviours, the present method estimated the overall crowd flow with the microscopic model without distinguishing the individuals. This simplification enabled the forecasting of much larger crowd flows, involving thousands of individuals, as confirmed by the numerical experiments.

A key concept in the present method is the introduction of latent parameters, which are assumed to underlie the actual complex behaviours and macroscopically govern the crowd flow tendency. In Experiment 2 in this study, the actual imbalance of the crowd flow at the exits was caused by the realistic following model; however, the resulting observed behaviour was considered as an exit preference with the single parameter $\theta _{1}$, and the different crowd flows were successfully forecasted by sequentially estimating the parameter. Similarly, the distributed evacuation departure tendency was reproduced by only estimating parameter $\theta _{2}$ without any assumption on the departure distribution. Behavioural uncertainty in real crowd flows such as in the evacuation departures had limited the use of agent-based simulations for real-time applications, and agent-based simulations are generally used for what-if analysis; hence, they have not been “live”^19^. The proposed method fills a gap towards live simulations and has potential to lead to real-time applications of agent-based simulations such as real-time evacuation guidance. Although the present approach was tested mainly against evacuation scenarios, the core concept of the method is expected to be useful for wide variety of crowd flows.

The availability of the density map observation assumed in this study is feasible in terms of both technical and privacy aspects, and the results of the performance test with limited observations in this study also support the applicability of the present method to real crowd problems. However, several challenges remain for more complex, real-world problems. The first concerns the agent design in the simulations constructing the estimator. The crowd flow considered in this study is rather complex when compared to the previous efforts, but the principal behavioural tendency is still simple. This enabled us to appropriately design the agent-based simulation with the latent parameters in the particle filter and to forecast the synthesised crowd with reasonable accuracy. In modelling real crowds, however, it is not always easy to consider the least but sufficient model that can capture the principal tendency of the crowd flow. Methods for finding such models from the data to produce a good approximation of the observation should be studied to consider the applications to complex real crowds. The second concerns the correction of the simulation. In the current method, although the observation data weighed the simulations in the particle filter to estimate the likely crowd state and latent parameters, the data were not used to directly modify the running variables in the simulations, such as the agent positions, even if the gap between the actual crowd and the simulation could be identified by the observation. As a result, the estimator would give poor forecasting if there was a large gap between the observations and the simulations in the estimation. In contrast to the synthesised data, because more gaps are expected between the actual observed behaviour and simulation in real applications, a correction in running simulations would be required to give better forecasting. These are not directly applicable to agent-based simulations; however, the techniques utilising observation to correct simulations have been studied, such as in the field of atmospheric science^32^, and should be useful reference. The presented method is still expected to be applicable to real but simple crowd flow problems; however, further studies discussed above will certainly expand the scope of using agent-based simulations in real-time.

## Methods

### Agent-based crowd simulation

We used an agent-based simulation to synthesise an observation and forecast the crowd flow. The simplified force-based model^4^, which was developed based on previous force-based models^33,34^, was employed in this study. The model considered the effects of the surrounding pedestrians by calculating the repulsive force, similar to general force-based models. However, the effects of the obstacles were considered not by calculating the forces from those obstacles, but by the movement rules referencing potential grids as a simplification. Consequently, the movements of the agents are described by the following equation (1):

1 $$\begin{aligned} \frac{\mathrm{d}{\mathbf {v}}_{i}}{\mathrm{d}t} = \frac{v^{0}_{i}\mathbf {e}^{0}_{i} - \mathbf {v}_{i}}{\tau _{\alpha }} + \sum _{i{\ne }j}\mathbf {F}_{ij} \end{aligned}$$

Here, the first term on the right hand side represents the driving force to reach a destination, consisting of the *i*-th desired velocity $v^{0}_{i}\mathbf {e}^{0}_{i}$, the current velocity $\mathbf {v}_{i}$, and the constant parameter $\tau _{\alpha }$, which is the relaxation time. The second term on the right hand side, the interaction force $\mathbf {F}_{ij}$ proposed in^34^, is considered and calculated by the following equation (2):

2 $$\begin{aligned} \mathbf {F}_{ij} = - {\nabla }_{\mathbf {r}_{ij}}(k{\tau ^{-2}}e^{{-\tau }/{\tau _0}}) \end{aligned}$$

where $\tau$ is the estimated time to collision, which is calculated using the relative displacement $\mathbf {r}_{ij} = \mathbf {r}_i - \mathbf {r}_j$ and the relative velocity $\mathbf {v}_{ij} = \mathbf {v}_i - \mathbf {v}_j$ between agents *i* and *j*, and *k* and ${\tau }_0$ are constants. As shown in a previous study^4^, the model can qualitatively and quantitatively reproduce interactions between agents and obstacles. Since the model has been validated against various fundamental diagrams, we used the same model parameter configuration presented in the original paper^4^.

### Crowd flow forecasting with sequential latent parameter estimation

Using the agent-based model and the synthetic observation data presented in the previous sections, we forecasted crowd flow by incorporating observation data into the simulation model. In this study, we propose a method to forecast crowd flow by sequentially estimating the latent simulation parameter governing the crowd flow with an agent state using a particle filter. Here, we assume that the transition of the crowd flow and its observation can be expressed by the nonlinear non-Gaussian state space model, which is described by the following equations (3) and (4):

3 $$\begin{aligned}&\mathbf {x}_{k} = f(\mathbf {x}_{k-1}, \mathbf {u}_k) \end{aligned}$$

4 $$\begin{aligned}&\mathbf {y}_{k} = h(\mathbf {x}_{k}, \mathbf {n}_k) \end{aligned}$$

where $\mathbf {x}_{k}$ and $\mathbf {y}_{k}$ are the state vector and the observation at time *k*. In this study, *f* and *h* are the nonlinear functions corresponding to the agent-based simulation and the transformation of a state into a density map (aggregate observation), respectively. $\mathbf {u}_k$ and $\mathbf {n}_k$ are the system and observation noise, respectively. The effect of randomised parameters in crowd simulation can be considered as system noise. Observation noise is not considered in this study.

Unlike previous studies using a particle filter with a microscopic agent-based model to estimate crowd flow^24,25^, the present method assumes that $\mathbf {x}_{k}$ includes not only the state of the agents (e.g., their position, velocity and movement state) but also the macroscopic simulation parameters $\varvec{\theta }_{k}$. In this study, we refer to $\varvec{\theta }_{k}$ as latent parameters that macroscopically govern the behavioural tendency of crowds. The method, rather than considering the parameters for each agent, assumes that the latent macroscopic parameters and the corresponding simple behavioural modes exist behind the observed complex crowd, and forecasts the crowd flow by sequentially adjusting the latent parameters. For the crowd flow in Experiment 1, we considered $\theta _{1}$ to represent the exit choice preference, i.e., agent *i* chooses the left exit and not the right exit if $U_{1}^{(i)} \le {\theta }_{1}$, where $U_{1}^{(i)}$ follows an uniform distribution $\mathcal {U}[0,1]$, i.e., $U_{1}^{(i)} \sim \mathcal {U}[0,1]$. In addition to $\theta _{1}$, a latent parameter $\theta _{2}$ controlling the evacuation departure is considered for the crowd flow in Experiment 2, i.e., agent *i* starts to move if $U_{2}^{(i)} \le {\theta }_{2}$, where $U_{2}^{(i)} \sim \mathcal {U}[0,1]$. The initial particles were generated with a random $\varvec{\theta }$ ranging from zero to one. Note that we did not include any information regarding the departure distribution and the follower model in the simulation model *f* to estimate the crowd. Therefore, the synthesised complex crowd movement in Experiment 2 was estimated using a simple agent-based model and the latent parameters $\theta _{1}$ and $\theta _{2}$.

The particle filter^35,36^ approximates the filtering distribution $p(\mathbf {x}_{k-1}|\mathbf {y}_{1:k-1})$ and the predictive distribution $p(\mathbf {x}_{k}|\mathbf {y}_{1:k-1})$ with the *N* particles $\{ \mathbf {x}^{(l)}_{k-1|k-1} \}^{N}_{l=1}$ and the predicted particles $\{ \mathbf {x}^{(l)}_{k|k-1}\}^{N}_{l=1}$ as the following equations (5) and (6):

5 $$\begin{aligned}&p(\mathbf {x}_{k-1}|\mathbf {y}_{1:k-1}) \simeq \frac{1}{N} \sum _{l} {\delta }\left( \mathbf {x}_{k-1} - \mathbf {x}^{(l)}_{k-1|k-1}\right) \end{aligned}$$

6 $$\begin{aligned}&p(\mathbf {x}_{k}|\mathbf {y}_{1:k-1}) \simeq \frac{1}{N} \sum _{l} {\delta }\left( \mathbf {x}_{k} - \mathbf {x}^{(l)}_{k|k-1}\right) \end{aligned}$$

where $\delta$ is the Dirac delta function. Afterwards, we can sequentially obtain the filtering distribution with Supplementary Algorithm S1. In this study, the weights of the particles, $\lambda ^{(l)}_k$, are calculated based on the sum of the absolute errors over the grids between the observed density map $\mathbf {y}_{k}$ and the simulated density map $\hat{\mathbf {y}}_{k}$, i.e., $\lambda ^{(l)}_k = 1 / \sum |\mathbf {y}_{k} - \hat{\mathbf {y}}_{k}|$, which is similar to the weighting method in data assimilation for an ocean model^37^. For the resampling algorithm, we employ residual systematic resampling^38^ (Supplementary Algorithm S2). To maintain the diversity of the particles after resampling, we add the Gaussian noise ${\epsilon }_{\theta }{\sim }\mathcal {N}(0,0.1^2)$ to each latent parameter at every 1 s, similar to roughening^35^. This noise helps the estimator explore possible states and follow the observation. With these algorithms, the state of the crowd and the latent parameters can be sequentially estimated based on the observations. This assimilation procedure is executed every 100 simulation steps, which corresponds to 1 s in the crowd simulation. The number of particles used in this study is 500. Hyperparameters such as the amplitude of the noise and the reasonable number of particles are determined in preliminary experiments.

## Supplementary Information

Supplementary Information.
