# Supplementary material for: Crowd flow forecasting via agent-based simulations with sequential latent parameter estimation from aggregate observation
Source: Sci Rep. 2022 Jul 1;12:11168. doi: 10.1038/s41598-022-14646-4 (PMC9249888; doi:10.1038/s41598-022-14646-4)
Supplement: Supplementary file 1 — Supplementary Information. [file 41598_2022_14646_MOESM1_ESM.pdf]

Supplementary Information

Crowd flow forecasting via agent-based simulations with sequential latent parameter estimation from aggregate observation

Fumiyasu Makinoshima[1,*] **and Yusuke Oishi**[1]

[1]Fujitsu Limited, Kawasaki, 211-8588, Japan
[*]Corresponding author: f.makinoshima@fujitsu.com

Crowd flow forecasting with sequential latent parameter estimation

The particle filtering algorithm and the resampling algorithm are descibed in Algorithm S1 and S2, respectively.

Algorithm S1 Particle filter

Generate initial distribution $\{\mathbf{x}_{0|0}^{(l)}\}_{l=1}^{N}$ with initial agent states and randomised latent parameters.
for $k = 1, \cdots, T$ **do**
  Obtain predictive distribution: $\{\mathbf{x}_{k|k-1}^{(l)}\}_{l=1}^{N}$ by calculating $\mathbf{x}_{k|k-1}^{(l)} = f(\mathbf{x}_{k-1|k-1}^{(l)}, \mathbf{u}_{k}^{(l)})$ for $l = 1, \cdots, N$
  Calculate the weights for the particles: $\lambda_k^{(l)} = p(\mathbf{y}_k|\mathbf{x}_{k|k-1}^{(l)})$ for $l = 1, \cdots, N$
  Calculate the normalised weights for the particles: $\beta_k^{(l)} = \lambda_k^{(l)} / \sum_L \lambda_k^{(L)}$ for $l = 1, \cdots, N$
  Resampling based on $\beta_k^{(l)}$ to obtain particles $\{\mathbf{x}_{k|k}^{(l)}\}_{l=1}^{N}$ approximating filtering distribution $p(\mathbf{x}_k|\mathbf{y}_{1:k}) \simeq 1/N \sum_l \delta(\mathbf{x}_k - \mathbf{x}_{k|k}^{(l)})$
end for

Algorithm S2 Residual systematic resampling

Generate a random number $U \sim \mathscr{U}[0, 1/N]$
for $l = 1, \cdots, N$ **do**
  Calculate the number of samples m for particle l: $m^{(l)} = \lfloor (\beta^{(l)} - U) \cdot N \rfloor + 1$
  Update U: $U = U + m^{(l)}/N - \beta^{(l)}$
end for
